# Tongue pressure profile training for dysphagia post stroke (TPPT): study protocol for an exploratory randomized controlled trial

**DOI:** 10.1186/1745-6215-14-126

**Published:** 2013-05-07

**Authors:** Catriona M Steele, Mark A Bayley, Melanie Péladeau-Pigeon, Shauna L Stokely

**Affiliations:** 1 Swallowing Rehabilitation Research Laboratory, Toronto Rehabilitation Institute, University Health Network, 550 University Avenue #12-125, Toronto, ON M5G 2A2, Canada; 2 University of Toronto, Toronto, Canada; 3 Bloorview Research Institute, Holland Bloorview Kids Rehab, Toronto, Canada

**Keywords:** Deglutition, Dysphagia, Penetration-aspiration, Pressure, Tongue

## Abstract

**Background:**

It is estimated that approximately 50% of stroke survivors will experience swallowing difficulty, or dysphagia. The associated sequelae of dysphagia include dehydration, malnutrition, and aspiration pneumonia, all of which have can have serious medical consequences. To improve swallowing safety and efficiency, alternative nutritional intake methods (for example, a feeding tube) or a modified diet texture (such as pureed foods or thickened liquids) may be recommended but these modifications may negatively affect quality of life. An alternative approach to treating dysphagia has emerged over the past few years, targeting stronger lingual muscles through maximal isometric pressure tasks. Although these studies have shown promising results, thin-liquid bolus control continues to be challenging for patients with dysphagia. Previous work investigating lingual pressures when healthy participants swallow has suggested that greater task specificity in lingual exercises may yield improved results with thin liquids.

**Methods/design:**

This is a small, exploratory randomized clinical trial being conducted with post-stroke patients 4 to 20 weeks after onset of dysphagia secondary to impaired lingual control. At enrollment, participants are randomly assigned to one of two treatment protocols, either tongue pressure profile training (TPPT) or the control treatment, tongue pressure strength-and-accuracy training (TPSAT). Each treatment protocol consists of 24 sessions of treatment over 8 to 12 weeks with monitoring of tongue pressure as well as a baseline and outcome videofluoroscopic swallowing study. Tongue pressure measures, videofluoroscopic measures, and functional outcome measures will be obtained following training of 60 participants (30 in each condition), to determine whether TPPT yields better outcomes.

**Discussion:**

This study will continue to explore options beyond tube feeding and modified diets for people with neurogenic dysphagia following stroke. Should the novel protocol, TPPT, prove to be more effective than the TPSAT protocol, this may influence standards of care and best practices for patients with dysphagia involving impaired thin-liquid control as a result of stroke.

**Trial registration:**

Clinicaltrials.gov http://NCT01370083NCT01370083

## Background

Drinking thin liquids is something that most of us take for granted, yet this task is one that many patients with dysphagia (swallowing impairment) cannot do safely. Instead, these individuals receive liquids in thickened form: thickened juice, thickened coffee, and even thickened water [1]. The literature tells us that patients dislike the taste and feel of thickened liquids and find that their thirst is not quenched. Patients on a diet with thickened liquids are prone to inadequate fluid intake and dehydration [2,3]. Many patients are noncompliant and drink thin liquids, despite documented risk of aspiration (that is, airway invasion) and its consequences [4]. Given these limitations, it is important that dysphagia researchers continue to pursue treatments with the potential to restore safe and functional thin-liquid swallowing in people with dysphagia.

In the past decade, tongue pressure resistance training has emerged as an innovative treatment for dysphagia. Robbins has shown that 8 weeks of intensive tongue pressure resistance training improves tongue strength in healthy seniors and those with both chronic and acute dysphagia following stroke [5,6]. In these studies, she had the participants complete an 8-week tongue-strengthening training protocol with an Iowa oral performance instrument (IOPI). Her findings included significantly improved isometric tongue pressures and swallowing pressures in all of the three populations that she studied (healthy participants, acute dysphagia, and chronic dysphagia). Although significant gains were made in airway protection, as judged by reduced scores on the penetration-aspiration scale [7], it is of ongoing concern that people with dysphagia after a stroke often have difficulty controlling the flow of thin liquids. Early arrival of a liquid bolus in the pharynx, secondary to poor oral control, represents a risk of penetration-aspiration (entry of material into the airway) [8].

Prior case series studies [9-11] have shown that patients who have completed a tongue pressure strength-and-accuracy resistance training protocol (TPSAT) showed improvements in tongue strength and aspiration. However, participants in these studies were not specifically recruited based on evidence of impaired liquid flow control, so the impact of the protocol on this feature could not be clearly elucidated. An analysis of patients treated to date in our lab suggests that approximately 30% of participants who complete the TPSAT protocol experience functional improvements in swallow response time for thin-liquid swallows. We speculate that strength-focused tongue pressure resistance tasks might lack sufficient task specificity to target improved liquid flow-control outcomes directly. We think that tongue pressure resistance training protocols would yield improved swallowing outcomes if they were modified to focus on tasks with similar pressure profiles (strength and timing) to those seen in healthy liquid swallowing.

To investigate this hypothesis, we have completed a study of tongue pressures (strength and timing) in healthy people, which shows that tongue pressures are released more slowly with thin liquids than with thick liquids [12]. This finding reveals active control of thin-liquid flow, and suggests that both the strength and timing of tongue pressure play a role in flow control. We believe that treatment outcomes would be better if tongue pressure resistance training protocols take both strength and timing into consideration. To this end, we have recently identified a subset of tongue pressure training tasks for which the strength and timing profile of tongue pressure onset and release is similar to that seen in liquid swallowing in healthy individuals [13]. We hypothesize that a treatment protocol will have better potential to yield favorable outcomes for thin-liquid flow control if it focuses on such tasks.

## Methods/design

### Study objectives

The primary objective of this study is to explore whether tongue pressure resistance training can be used to improve impairments in thin-liquid flow control for individuals with neurogenic dysphagia. To measure thin-liquid flow control, we will calculate the duration that a thin liquid is located in the pharynx prior to the onset of hyolaryngeal movement for airway protection; this measure is also known as the swallow response time [8]. Swallow response times in aspirating stroke patients have recently been reported to be significantly longer (620 ms ± standard error of 160 ms) than those seen in nonaspirating patients (20 ms ± 100 ms) [8]. In this study, we want to determine whether TPPT, which addresses both timing and amplitude issues in tongue pressure generation, yields better functional outcomes with respect to swallow response time than strength-and-accuracy focused treatment. The ultimate goal of this work is to develop interventions that restore functional swallowing ability in individuals who are unable to swallow thin liquids safely.

### Study design

This study is a small, exploratory randomized trial comparing treatment outcomes for two tongue pressure resistance training protocols: TPPT focuses on tongue pressure strength and timing, whereas TPSAT emphasizes target accuracy during strength tasks. Both protocols build on recent tongue pressure resistance training studies, which emphasized load and intensity, and are informed by physiological principles of muscle training through exercise.

Each training protocol consists of 24 intervention sessions, in addition to a baseline measurement session, a baseline videofluoroscopic swallowing study (VFSS), and an outcome VFSS. The VFSS outcomes will be measured using a series of three teaspoon-sized boluses of thin-liquid barium (Liquid Polibar diluted with water to a 22% w/v concentration).

**Figure 1:**
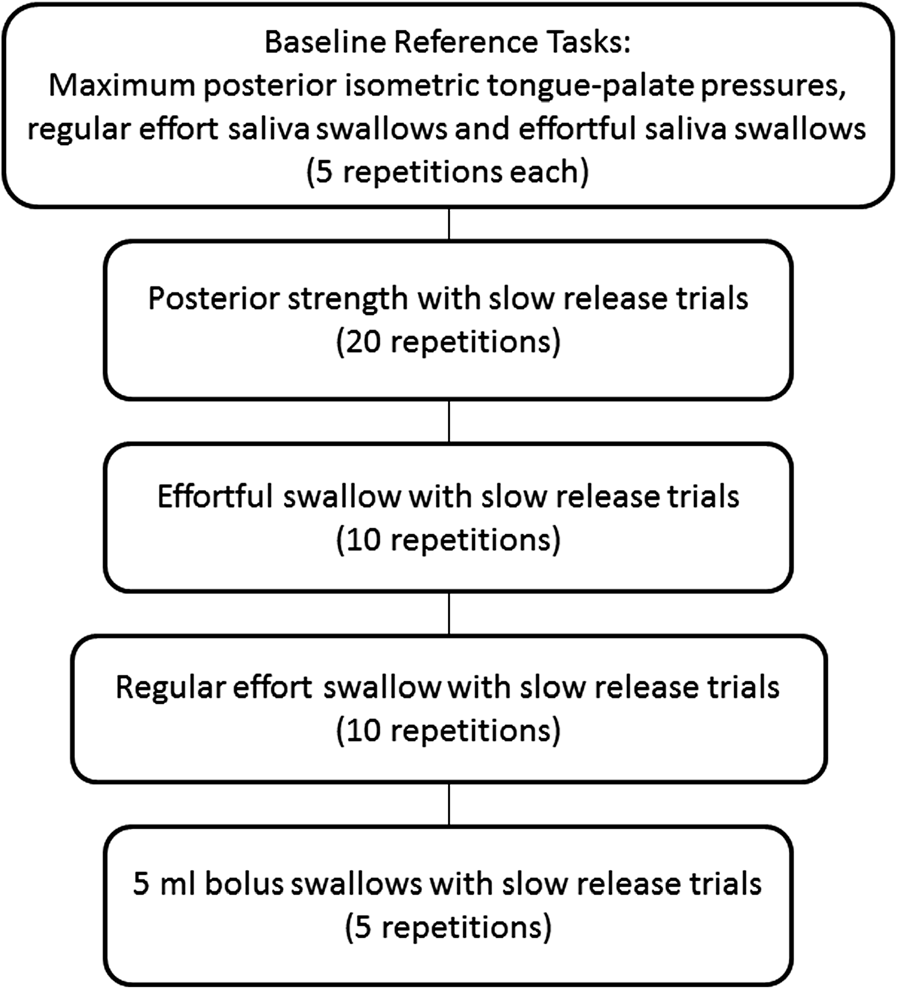
Outline of a tongue pressure profile training session.

**Figure 2:**
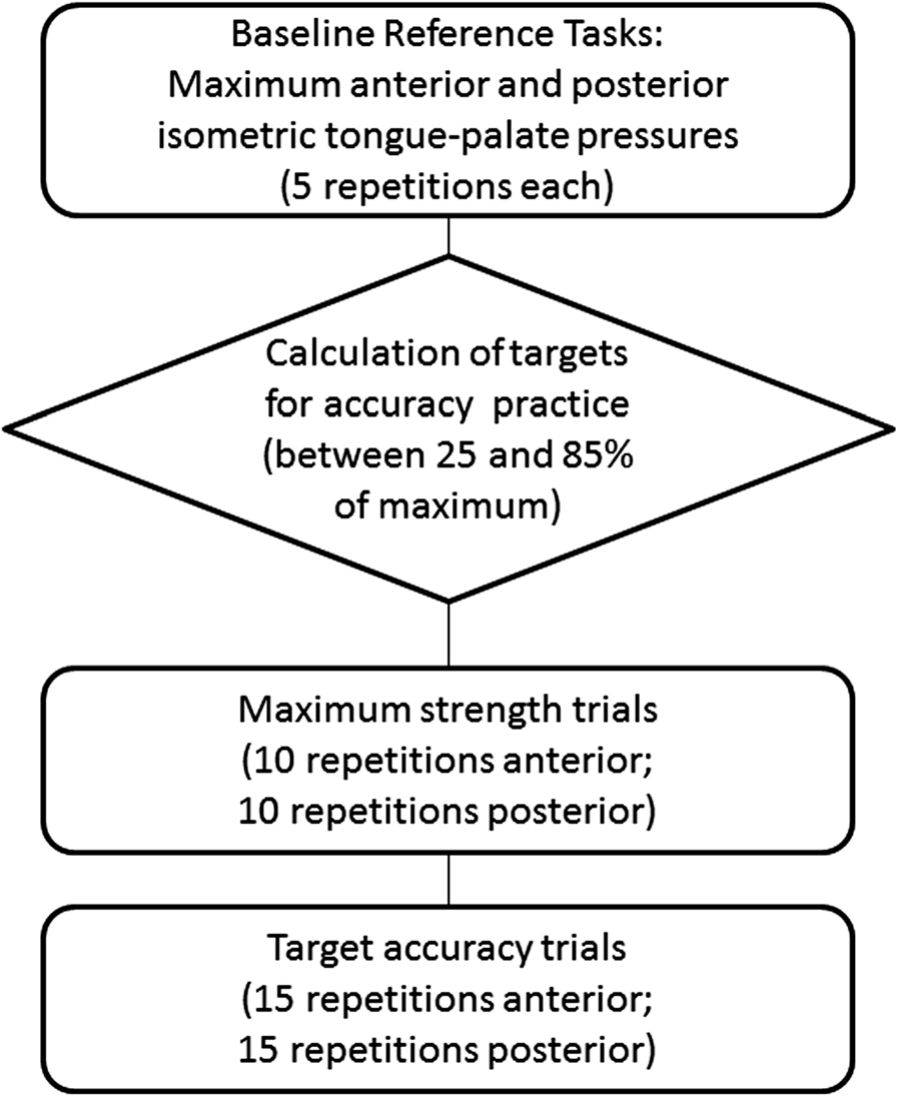
Outline of a tongue pressure strength-and-accuracy training session.

Each training program will be delivered as two or three sessions per week on nonconsecutive days, commencing directly after enrollment. The outlines of the TPPT and TPSAT sessions are illustrated in Figures 1 and 2, respectively. Tongue-palate pressure tasks are performed with the IOPI bulb placed in both an anterior position (that is, with the flat end of the bulb positioned immediately behind the upper incisors) and a posterior position (that is, with the flat end of the bulb lined up with the first molar tooth). Although the tasks vary between the two protocols, the total number of exercise repetitions is held constant between the two protocols at 60 exercises per session. For the slow pressure release tasks in the TPPT protocol, the instruction is for the participant to release pressure gradually from a peak to a resting value, spanning at least a 3-second interval. The bolus-swallowing tasks included in the TPPT protocol involve a series of five discrete swallows, each 5 ml in volume, of the thinnest liquid consistency (either thin, nectar- or honey-thick liquid) that can be safely tolerated by the patient, as determined by the intake VFSS examination. A rest period of at least 30 seconds is provided between each swallow.

With the exception of the bolus trials in the TPPT protocol, all tasks are performed using an IOPI. We have developed a computer interface that displays the IOPI waveform in real time on a computer-displayed graph to give visual biofeedback to the participant for all tasks.

All enrollment procedures, screening and training sessions will be conducted by a registered speech-language pathologist or a support worker (for example, a Communicative Disorders Assistant) under the direct or indirect supervision of a speech-language pathologist. All VFSS assessments will be conducted by a speech-language pathologist in conjunction with a radiation technologist.

### Study sites

This study is being conducted in two publicly funded rehabilitation centers in Ontario, Canada: the Toronto Rehabilitation Institute –University Health Network and the Kitchener-Waterloo Local Health Integration Network #3. Each of these settings has a neurorehabilitation program that provides inpatient and outpatient services.

### Ethical approval

This research protocol was reviewed and approved by the Toronto Rehabilitation Institute Research Ethics Board (#10-027), and the Tri-Hospital Research Ethics Board for Cambridge Memorial Hospital, Grand River Hospital and St. Mary’s General Hospital (#2012-0474).

### Study population

The participants enrolled in this study will have a primary diagnosis of stroke, as well as dysphagia presenting with prolonged swallow response time for thin liquids. Impaired swallow response time will be operationally defined as the presence of thin liquid in the pharynx (that is, below the shadow of the ramus of the mandible) for >350 ms prior to the onset of hyolaryngeal excursion. This threshold value falls halfway between the reported 95% confidence intervals for non-aspirating and aspirating stroke patients in a recent study [8]. We will measure this parameter on 5 ml swallows of thin-liquid barium using a cued swallow instruction. Participants must be patients (inpatient or outpatient) in one of the two participating rehabilitation settings to be enrolled in the study. Additional inclusion and exclusion criteria are detailed.

#### Inclusion criteria

To be included in the study, each participant must be able to understand English, be able to follow directions, and be able to tolerate oral trials under the supervision of a therapist. This is likely to exclude individuals with brainstem strokes, for whom swallowing is often profoundly impaired and oral trials are often unsafe. Additionally, to control for spontaneous recovery, enrollment into the study must occur at least four weeks after dysphagia onset and not more than 20 weeks following dysphagia onset. Within this enrollment window, a VFSS must be completed and recorded. The VFSS recording will then be scored by a speech-language pathologist to determine whether poor bolus control is present, as indicated by swallow response times >350 ms on teaspoon-sized volumes of thin-liquid barium. This will confirm eligibility to participate.

#### Exclusion criteria

Participants will be excluded for safety reasons, if they are pregnant, have any known allergies to chemicals, latex, or materials commonly found in a dentist’s office, or have an *in-situ* tracheostomy. Additional exclusion criteria are applied to ensure that the data are strictly measuring dysphagia in stroke patients only. These criteria are: no history of major surgery to the head, neck or mouth (other than routine tonsillectomy or previous tracheostomy); no known history of progressive neurological disorder (for example, Parkinson’s disease, multiple sclerosis); and no current or concurrent use of oral motor exercises targeted at improving swallowing.

Note that the participant’s diet is neither an inclusion nor an exclusion criterion; participants may be on an oral or non-oral diet with or without texture modifications or recommended compensatory strategies and participate in the study provided they meet the inclusion criteria. Furthermore, the participant’s recommended diet texture can be changed at any time during the protocol, provided it is clinically warranted.

### Enrollment

Enrollment is currently in progress; the targeted completion date is 2014.

### Randomization

During the intake procedure, participants will be randomly assigned to either the TPPT or TPSAT treatment conditions using a sequence generated by http://www.randomizer.org. Randomization will be concealed using sequentially numbered, opaque sealed envelopes.

### Blinding

It is not possible to blind the participants or treating clinicians to the condition given the protocol differences in the two arms of the trial. All data analysis, including treatment outcome, will be blinded. For VFSS rating, individual swallow clips for the first three teaspoon-sized boluses of 22% w/v thin-liquid barium will be spliced from the recordings for each participant, and arranged in a randomized order in a master rating set. Two blinded speech-language pathologists will review and rate these recordings.

### Outcome measures

Three types of data will be collected in this study: tongue pressure parameters, measures of swallow response time from videofluoroscopy, and measures of swallowing safety from videofluoroscopy. We will collect baseline and post-treatment pressure parameters (amplitude, duration, and slope) for the rise and release phases of tongue pressure events during three reference tasks (non-effortful saliva swallows, effortful saliva swallows, and posterior maximum isometric pressure tasks).

Videofluoroscopic measures will be collected at baseline and after treatment using a standardized videofluoroscopy protocol recorded on a KayPentax Digital Swallow Workstation at 30 frames per second. Two parameters will be rated for each spliced 5 ml thin-liquid barium (22% w/v) swallow:

1. Swallow response time (that is, the time difference between the arrival of the bolus head at the mandibular ramus and the onset of hyolaryngeal excursion, calculated in milliseconds, based on the time code of the video recording.

2. A measure on the eight-point penetration-aspiration scale [7], which determines the severity of and response to entry of the bolus into the airway.

The primary outcome variable of interest will be a measure of the participant’s average swallow response time across a series of three thin-liquid swallows (each 5 ml in volume). The targeted outcome will be an average swallow response time value shorter than 350 ms.

### Sample-size calculation

Our prior study of treatment outcomes from the TPSAT suggests that one-third of patients in the control group are likely to exhibit improvement in the primary outcome variable (average swallow response time above vs. below 350 ms). A sample-size calculation was conducted using Study Size Software 2.0, modeling the expectation (in a 2 × 2 contingency table) that the TPPT treatment will yield a greater rate of improvement, that is, in at least 66% of participants, than the current 30% response rate seen in cases accrued in our lab in historical studies. Under these assumptions, a sample-size calculation shows that 29 participants per group are needed to demonstrate a significant difference in rate of improvement at a *P* value of 0.05 and a power of 0.8. Therefore, we have established a targeted sample size of 60 participants (30 participants per protocol).

### Data processing and analysis

The proposed analysis of differences in pressure measures will be a 2 (group) × 3 (task) × 2 (measurement point) repeated measures mixed-model analysis of variance. We will perform this analysis using SPSS’s mixed modeling procedure, which will allow us to model the covariance structure of the data accurately.

From the VFSS measures, the primary measure of interest will be a binary reduction of the participant’s mean swallow response time measure (less than or more than 350 ms). We will also record whether or not the participant’s worst penetration-aspiration scale score across the series of three teaspoon-sized boluses of thin-liquid barium is less than or more than 3, representing material remaining above and inside or below the supraglottic space, respectively, as the measure of swallowing safety. Two-by-two tables with chi-square tests will be used to test for differences in the proportion of participants in each treatment group with impaired versus normal swallow response time at the post-treatment VFSS, and also the proportion in each group who display impaired swallowing safety. Odds ratios for reduction in swallow response time and improved swallowing safety will be computed.

## Discussion

Individuals suffering from dysphagia have limited treatment options. Alternative feeding methods such as PEG-tubes (percutaneous endoscopic gastrostomy) or modifying the diet texture may not always be appropriate or desirable, given the potential risks and implications for reduced quality of life. Unlike these two options, this protocol actively works to rehabilitate the tongue’s ability to control thin boluses, with the goal of the individual being able to consume thin liquids safely. This study has been designed to build on previous work examining tongue pressure patterns in healthy controls to identify tasks that might help a post-stroke clinical population. If found to be effective, the TPPT protocol could inform the types of tasks required to optimize dysphagia therapy and recovery for individuals with tongue impairment following stroke. Future research might identify the optimal timing, duration and frequency for TPPT interventions and the implications (for example, cost, quality of life, overall health) of TPPT as compared with PEG-tube feeding or modified texture diets.

## Trial status

Enrollment is currently in progress; the targeted completion date is 2014.

## Abbreviations

IOPI: Iowa oral performance instrument; PEG: percutaneous endoscopic gastrostomy; TPPT: tongue pressure profile training; TPSAT: tongue pressure strength-and-accuracy training; VFSS: videofluoroscopic swallowing study.

## Competing interests

The authors declare that they have no competing interests.

## Authors’ contributions

CMS is the principal investigator for this trial. She designed the protocol and is responsible for oversight of all aspects of the study. MAB is the physician co-investigator for this study. He is responsible for oversight of patient eligibility at the primary data collection site and has contributed to study design. MP-P is a research associate working on this study. She has designed the software interface used for biofeedback during data collection, and is responsible for clinician training and data processing. SLS is a research associate and speech-language pathologist working on this study. She is involved in data collection and clinician training and contributed to manuscript preparation and review. All authors read and approved the final manuscript.

## References

[B1] Cichero JAY Atherton M Bellis-Smith N Suter M Texture-modified foods and thickened fluids as used for individuals with dysphagia: Australian standardised labels and definitions Nutr Diet 2007 64 Suppl. 2 S53 S76

[B2] Finestone HM Foley NC Woodbury MG Greene-Finestone L Quantifying fluid intake in dysphagic stroke patients: a preliminary comparison of oral and nonoral strategies Arch Phys Med Rehabil 2001 82 12 1744 1746 10.1053/apmr.2001.27379 11733894

[B3] Robbins J Gensler G Hind J Logemann JA Lindblad AS Brandt D Baum H Lilienfeld D Kosek S Lundy D Dikeman K Kazandjian M Gramigna GD McGarvey-Toler S Miller Gardner PJ Comparison of 2 interventions for liquid aspiration on pneumonia incidence: a randomized trial Ann Intern Med 2008 148 7 509 518 10.7326/0003-4819-148-7-200804010-00007 18378947 PMC2364726

[B4] Colodny N Dysphagic independent feeders’ justifications for noncompliance with recommendations by a speech-language pathologist Am J Speech Lang Pathol 2005 14 1 61 70 10.1044/1058-0360(2005/008) 15962847

[B5] Robbins J Kays SA Gangnon RE Hind JA Hewitt AL Gentry LR Taylor AJ The effects of lingual exercise in stroke patients with dysphagia Arch Phys Med Rehabil 2007 88 2 150 158 10.1016/j.apmr.2006.11.002 17270511

[B6] Robbins J Gangnon RE Theis SM Kays SA Hewitt AL Hind JA The effects of lingual exercise on swallowing in older adults J Am Geriatr Soc 2005 53 9 1483 1489 10.1111/j.1532-5415.2005.53467.x 16137276

[B7] Rosenbek JC Robbins J Roecker EB Coyle J Wood JL A penetration-aspiration scale Dysphagia 1996 11 93 98 10.1007/BF00417897 8721066

[B8] Power ML Hamdy S Goulermas JY Tyrrell PJ Turnbull I Thompson DG Predicting aspiration after hemispheric stroke from timing measures of oropharyngeal bolus flow and laryngeal closure Dysphagia 2009 24 3 257 264 10.1007/s00455-008-9198-4 19252944

[B9] Yeates EM Molfenter SM Steele CM Improvements in tongue strength and pressure-generation precision following a tongue-pressure training protocol in older individuals with dysphagia: three case reports Clin Interv Aging 2008 3 4 735 747 19281066 10.2147/cia.s3825 PMC2682406

[B10] Steele C Molfenter S Bailey G Oshalla M Yeates E Tongue-pressure strength and accuracy training (TPSAT) for thin liquid dysphagia Dysphagia 2011 26 442 10.3109/17549507.2012.752864 PMC3793268 23336825

[B11] Steele CM Bailey GL Cliffe Polacco R Hori S Oshalla M Molfenter SM Yeates EM Outcomes of tongue-pressure strength and accuracy training for dysphagia following acquired brain injury Int J Speech Lang Pathol 2013 10.3109/17549507.2012.752864 PMC3793268 23336825

[B12] Steele CM Bailey GL Molfenter SM Tongue pressure modulation during swallowing: water vs. nectar-thick liquids J Speech Lang Hear Res 2010 53 2 273 283 10.1044/1092-4388(2009/09-0076) 20008678

[B13] Steele CM Bailey GL Molfenter SM Yeates EM Pressure profile similarities between tongue resistance training tasks and liquid swallows J Rehabil Res Dev 2010 47 7 651 660 10.1682/JRRD.2009.05.0068 21110261

